# Effects of COVID-19 prevention procedures on other common infections: a systematic review

**DOI:** 10.1186/s40001-021-00539-1

**Published:** 2021-07-03

**Authors:** Omid Dadras, Seyed Ahmad Seyed Alinaghi, Amirali Karimi, Mehrzad MohsseniPour, Alireza Barzegary, Farzin Vahedi, Zahra Pashaei, Pegah Mirzapour, Amirata Fakhfouri, Ghazal Zargari, Solmaz Saeidi, Hengameh Mojdeganlou, Hajar Badri, Kowsar Qaderi, Farzane Behnezhad, Esmaeil Mehraeen

**Affiliations:** 1 School of Public Health, Walailak University, Nakhon Si Thammarat, Thailand; 2 Iranian Research Center for HIV/AIDS, Iranian Institute for Reduction of High Risk Behaviors, Tehran University of Medical Sciences, Tehran, Iran; 3 School of Medicine, Tehran University of Medical Sciences, Tehran, Iran; 4 School of Medicine, Islamic Azad University, Tehran, Iran; 5 School of Medicine, Iran University of Medical Sciences, Tehran, Iran; 6 Department of Nursing, Khalkhal University of Medical Sciences, Khalkhal, Iran; 7 Department of Pathology, Urmia University of Medical Sciences, Urmia, Iran; 8 School of Health, Guilan University of Medical Sciences, Rasht, Iran; 9 Kermanshah University of Medical Sciences, Kermanshah, Iran; 10 Department of Virology, School of Public Health, Tehran University of Medical Sciences, Tehran, Iran; 11 Department of Health Information Technology, Khalkhal University of Medical Sciences, 1419733141 Khalkhal, Iran

**Keywords:** COVID-19, SARS-CoV-2, Infection, Prevention

## Abstract

**Introduction:**

Since the outbreak of the severe acute respiratory syndrome coronavirus-2 (SARS-CoV-2) began, necessary measures to prevent virus transmission and reduce mortality have been implemented, including mandatory public use of masks, regular hand-sanitizing and hand-washing, social distancing, avoidance of crowds, remote work, and cancellation of public events. During and after the introduction of COVID-19 lockout, we performed a systematic review of available published literature to investigate the incidence of seasonal influenza and other respiratory viral infections.

**Methods:**

PubMed, Embase, Web of Science, Scopus, Science Direct, Google Scholar, Research Gate, and the World Health Organization databases and websites were systematically searched for original studies concerning the impact of COVID-19 prevention means and measures on other common respiratory infectious diseases during the pandemic published by March 2021.

**Results:**

The findings showed that the adherence to health protocols to prevent COVID-19 could help to reduce the incidence of other infectious diseases such as influenza, pneumonia, and Mycobacterium tuberculosis.

**Conclusion:**

The implemented prevention measures and protocols might have reduced the incidence of influenza and some other common respiratory infections. However, controversies exist on this matter and future large population-based studies might provide further information to address these controversies.

## Introduction

Since the beginning of the severe acute respiratory syndrome coronavirus-2 (SARS-CoV-2) outbreak, necessary measures have been taken to prevent the virus transmission and reduce mortality, such as mandatory public use of mask, regular hand-sanitizing and hand-washing, remote work, social distancing, avoid gatherings, and cancellation of public events [1]. Limiting contact is a strategy that aims to reduce both the frequency and duration of contacts, lowering the basic reproduction number, R0, or the average number of people to whom one case transmits the disease during his or her incubation period [2].

As a control measure, China was the first country to implement a regional lockdown of cities in Hubei province. Wuhan, the largest city in Hubei province, with a population of over 14 million people, was subjected to a 76-day lockdown. Other countries, including Italy (provinces of Lombardy and Veneto), Spain, Russia, India, and the Philippines, later used similar lockdowns, with durations ranging from as little as 4 days in Turkey to as long as nearly a year in Qatar (3Oraby). Studies have shown that strategies have been effective in preventing the spread of the disease and reducing the incidence and mortality rates [2–4].

It is hypothesized that SARS-CoV-2 measures may also be effective in reducing other respiratory infectious diseases, such as seasonal influenza, outpatient pneumonia, scarlet fever, and severe acute respiratory illness (SARI) [5, 6]. Findings of recent study conducted in New Zealand showed that after 9 months of lockdown, the incidence of influenza decreased 79-fold. They have also reported a significant reduction in the incidence of other respiratory viruses during post-lockdown in comparison with the same time in the past year [7]. A reduction in the number of people infected with the influenza virus in 2020 compared to the previous year was also observed in a study from Japan [8]. Here, we assessed the reduction of the seasonal influenza virus incidence and respiratory viral infections during and after the implementation of COVID-19 lockdown.

## Methods

### Design

We systematically searched PubMed, Embase, Web of Science, Scopus, Science Direct, Google Scholar, Research Gate, and the World Health Organization databases and websites. After conducting the search protocol, two researchers performed a two-step screening process. First, they screened the title/abstract of the retrieved records. Then, the selected articles in the first step underwent a full-text screening and the eligible studies were identified.

### Research question

We aimed to answer the following main question:

- What was the impact of COVID-19 prevention means and measures on other common respiratory infectious diseases during the pandemic?

### Search strategy

We included the final search entry [C] for this systematic review:

A. [Viral infection] (Title/Abstract) OR [SARS] (Title/Abstract) OR [Respiratory tract infection] (Title/Abstract) OR [Interstitial lung diseases] (Title/Abstract) OR [Influenza] (Title/Abstract) OR [Inflammation] (Title/Abstract)
B. [Coronavirus] (Title/Abstract) OR [COVID-19] (Title/Abstract) OR [SARS-CoV-2] (Title/Abstract) OR [Corona] (Title/Abstract) OR [COVID] (Title/Abstract)OR [Coronavirus infection] (Title/Abstract)OR [Pandemic] (Title/Abstract)
C. [A] AND [B]

### Eligibility criteria

English articles relevant to our research question from the start of the pandemic (December 2019) until March 2021 were included.

The exclusion criteria were as follows:

- Review articles, editorial, commentaries, opinions, or any studies with no original data
- Ongoing projects (e.g., articles discussing the protocol of a future study)
- Non-human (e.g., animal and laboratory) studies
- Duplicated results in databases.

### Data collection

Two researchers read the full texts of the eligible articles and extracted the relevant findings. The collected data were utilized to construct the results section.

## Results

**Fig. 1:**
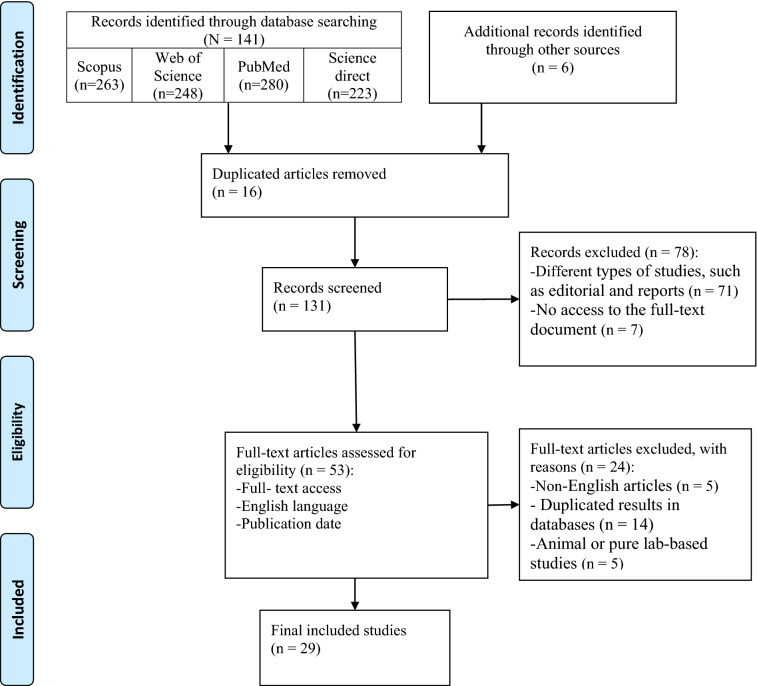
PRISMA 2009 flow diagram

In this study, 147 documents were identified using a systematic search strategy. After a primary review of retrieved articles, 16 duplicates were removed, and the title and abstract of the remaining were reviewed. After applying the selection criteria, 102 articles were excluded, and only 29 articles met the inclusion criteria and were included in the final review (Fig. 1). These studies were conducted in Taiwan (*n* = 9), between countries (*n* = 4), Singapore (*n* = 3), Japan (*n* = 2), South Korea (*n* = 2), China (*n* = 2), Hong Kong (*n* = 1), Mexico (*n* = 1), the Netherlands (*n* = 1), the European region (*n* = 1), Israel (*n* = 1), Pakistan (*n* = 1), and the USA (*n* = 1) and explored the effect of COVID-19 prevention measures on other respiratory infectious diseases. From 29 included studies, 25 were related to influenza, four related to Mycobacterium tuberculosis, four related to pneumonia, three related to rhinovirus, three related to other coronaviruses, three related to enterovirus, two related to adenovirus, and only one study was related to several respiratory infections.

The present study results showed that adherence to the COVID-19 preventive measures and protocols could be protective and reduce the incidence of some other respiratory infectious diseases such as influenza, pneumonia, and Mycobacterium tuberculosis.

## Discussion

SARS-CoV-2, a novel coronavirus responsible for COVID-19, was first reported in China in late 2019 and then spread out rapidly worldwide [35]. COVID-19 attracted worldwide attention as an international public health emergency and the first pandemic caused by a coronavirus [36]. Many attempts were made to control the situation, such as border controls, social distancing, community education, lockdown, compulsory wearing of masks in public, and school closures [37–40]. These public health measures not only reduced SARS-CoV-2 transmission, but might also reduce transmission of other endemic viral respiratory infections [10]. In this study, we gathered and reported the findings that support or reject this hypothesis.

One of the respiratory viruses is influenza. Influenza is one of the most common respiratory infectious diseases and a highly contagious airborne disease that occurs as seasonal epidemics and manifests as an acute febrile illness with variable degrees of systemic symptoms [41]. Public health interventions and changes (such as stay-at-home orders, movement restrictions, face mask use, and school closures) in response to the SARS-CoV-2 outbreak unintentionally led to the reduction of influenzas spread in 2020 [10, 11]. Singh S et al. have noted an initial increase in influenza testing in southeastern Wisconsin followed by a dramatic decline in detection of seasonal influenza coinciding with the outbreak of SARS-CoV-2 [27]. They found that the decline in influenza detection during the 2019–2020 seasons could have been due to less testing for influenza, which may have happened if clinicians had started testing preferentially for SARS-CoV-2 during this period. However, they also responded to this by stating that influenza testing was probably much higher or the same during this period compared to previous years. As influenza and SARS-CoV-2 are symptomatically indistinguishable, they concluded that it is highly unlikely that patients with influenza decided to avoid seeking medical care [27]. Young G et al. have also found that implementing public health measures in the early fall, warranted by the first signs of either influenza or COVID-19 outbreaks, may help to reduce the transmission of both respiratory illnesses. However, they were concerned about the potential impact of a second wave of COVID-19 during the upcoming influenza season in the upcoming autumn [34].

Following a period of reduction in COVID-19 transmission over the summer, most countries observed a rise in COVID-19 cases and initiated using various preventive public health and social measures [32]. The continuation or re-implementation of several public health and social measures led to an overall reduction in circulating influenza viruses again during the winter months compared with previous seasons, particularly considering the lower basic reproduction number (R0) of influenza compared with SARS-CoV-2 [32]. Singh S et al. showed that convincing the public to change its lifestyle through policy and information—as was gained at the outset of the SARS-CoV-2 outbreak—could again reduce the impact of a “double hit” at fall and winter. These efforts saved lives directly by reducing the incidence of seasonal influenza and SARS-CoV-2, and indirectly by reducing the burden on the health system [27].

Nevertheless, this theory might not be entirely correct, and some findings are controversial. For instance, the number of documented Influenza and pneumonia deaths in the US was higher in 2020 than in 2019. Documented influenza and pneumonia deaths increased by 7.5% in 2020, although the number of influenza and pneumonia deaths was lower in 2020 than in 2017 and 2018 [42]. However, there is a concern that wrong death certificates might alter the reality of the situation, especially in the pandemic where the high burden might have resulted in wrong findings and documentation, especially for diseases with similar manifestations such as influenza and COVID-19 [43, 44].

Besides influenza, studies show a meaningful reduction in the activity and reported cases of other respiratory germs such as Mycobacterium tuberculosis, pneumonia, rhinovirus, enterovirus, adenovirus, and several other respiratory infectious diseases mentioned in Table 1 [5, 19, 20, 22, 25, 26, 29]; however, many of them are scarcely investigated. For example, measles cases in Taiwan were reported to be zero after the pandemic, possibly owing to the implemented precautions during the pandemic [12]. In supporting the above-mentioned conclusion, Lai CC et al. have noticed the significant decline of tuberculosis activity during the COVID-19 outbreak in Taiwan. Droplet aerosol precaution and prevention measures may offer success in containing SARS-CoV-2 transmission and collateral benefits in controlling tuberculosis [20]. However, Komiya K et al. have another idea about these statistics and explain them in another way. They think that the statistical decline in tuberculosis incidence following the COVID-19 outbreak is likely to have been influenced by the decreased number of tests for M. tuberculosis and may not reflect the true incidence of tuberculosis in Japan. They justified this by stating that the reactivation of tuberculosis among the elderly cannot be controlled by short-term measures for preventing SARS-CoV-2 transmission [18].

**Table 1:** Induced infectious diseases during COVID-19

ID	The first author (reference)	Type of study	Country	Study population	Influenza A, B	Rhinovirus	Enterovirus	Adenovirus	Mycobacterium tuberculosis	Pneumococcus	Other coronaviruses	Other
1	Adlhoch [9]	Editorial	European region	Interseasonal weeks 21–39 2014–2020	*	*						
2	Arellanossoto [10]	Cross-sectional	Mexico	Data from 2016 to 2017 season to the 2019–2020 season from epidemiological week 40 to week 30	*							
3	Chan [11]	Cross-sectional	Taiwan	Data from 25 calendar weeks of the influenza season for four years (2016–20)	*					*		
4	Chen [12]	Cross-sectional	Taiwan	Extracted the reported case numbers for measles between January and August from 2015 to 2020 for comparison								measles
5	Cowling [13]	Cohort	Hong Kong	Data obtained from 60 general outpatients clinics/and from public hospitals	*							
6	Enserink [14]	Cross-sectional	Netherlands	Data obtained from nursing homes	*							
7	Galvin [15]	Short communication (editorial)	Taiwan	–	*							
8	Hsu [16]	Letter to editor	Taiwan	Data from influenza season 2018 to 2019 and 2019 to 2020	*							
9	Itaya [17]	Cross-sectional	11 countries	Extraction of weekly reports of seasonal influenza data from 2014–2015 season to the 2019–2020 season from epidemiological week 40 to week 10	*							
10	Komiya [18]	Letter to editor	Japan	Data from acid-fast bacterial cultures from January to May in 2020 with the same period in 2017, 2018 and 2019					*			
11	Kuo [6]	Cross-sectional	Taiwan	Comparison of data from the first 12 weeks of 2020 with data from the same period of 2019	*							Varicella
12	Lai [19]	Letter to editor	Taiwan	Data obtained from NIDs for infectious disease between January and October in 2019 and 2020 for comparison	*				*	*		11 infectious disease:varicella/legionella. Mumps/Q fever/meningococcal meningitis/hanta virous/measles/rubella/pertussis (14 diseases totally)
13	Lai [20]	Letter to editor	Taiwan	Data on infectious disease obtained (first 20 calendar weeks of 2020 and compared with the same period of 2017, 2018 and 2019					*			
14	Lee [21]	Cross-sectional	Korea	National influenza surveillance data were compared between 7 sequential seasons	*							
15	Lee [5]	Cross-sectional	Taiwan	Data obtained from 181 hospitals (a comparison the number of outpatient visits for influenza, pneumonia, enterovirus infection and scarlet fever (week 40 in 2019 through week 18 in 2020)	*		*					Scarlet fever /pneumonia
16	Lim [22]	Letter to the editor	Singapore	Routine infectious disease surveillance—urinary streptococcal antigen tests + notifications submitted to the Ministry of Health for IPD, 2010 to 2020 (e-weeks 1–27)			*			*		
17	Noh [23]	Cross-sectional	Korea	Data obtained from Korea Centers for Disease Control and Prevention (KCDC) From Sep 1st, 2019–2018 to Apr 2020	*							
18	Olsen [24]	CDC Report	USA, Australia, Chile, South Africa	a) Data obtained from 300 U.S. clinical laboratories—WHO Collaborating Laboratories System + National Respiratory and Enteric Virus Surveillance System, Sep 29th, 2019 to Feb 29th, 2020 Vs. Mar 1st to May16th, 2020 b) Laboratory data from clinical and surveillance platforms reported from Australia, Chile, and South Africa to WHO’s FluNet platform, April–July (weeks 14–31) 2017–2020	*							
19	Oster [25]	Letter to the editor	Israel	Patients hospitalized at the Hadassah Medical Center during April–August 2020 vs. total numbers observed in the previous 3 years	*			*			*	Parainfluenza 1,2,3. RSV. Mycoplasma pneumonia. Bordetella pertussis
20	Rana [26]	Letter to the editor	Pakistan	Data based on the surveillance records available at NIH Islamabad. Data for non-infectious respiratory diseases obtained from the chest care clinic Islamabad, 2019–2020	*				*	*	*	RSV, measles, rubella, pertussis, diphtheria, EBV, H. influenza, VZV
21	Sakamoto [8]	Research	Japan	Data obtained from the National Institute of Infectious Diseases Japan from 5000 sentinel centers between 2014 and Mar 15th, 2020	*							
22	Singh [27]	Brief report	USA (Wisconsin)	Data from 2015–2020 retrieved from academic health system laboratory in southeastern Wisconsin	*							
23	Soo [28]	Cross-sectional	Singapore	Routine sentinel surveillance data on ILI from a national network of primary care clinics and the National Public Health Laboratory, comparison of influenza activity between epidemiologic weeks 1–4 and weeks 5–9 of 2020 Vs. 2016–2019	*							
24	Tan [29]	Letter to the editor	Singapore	Laboratory confirmed (16-target PCR on oropharyngeal specimen) community-onset respiratory viruses that were admitted to Singapore general hospital from Jan 1st, 2017 to Jul 1st, 2020	*	*	*	*			*	RSV A/B, Parainfluenza viruses 1–4, metapneumovirus, human coronavirus OC43/229E/NL63, human bocavirus 1–4
25	Wong [30]	Cross-sectional	Hong Kong, China	Surveillance data obtained from the Hong Kong Government Centre for Health Protection between 2019 and 2020	*							
26	Wu [31]	Letter to the editor	China	Data obtained from the Chinese National Influenza Center from 2019 to 2020	*							
27	Wu [32]	Letter to the editor	China	Data obtained from the Chinese National Influenza Center, Influenza-like illness samples, 2020	*							
28	Wu [33]	Letter to the editor	Taiwan	National data of critical/severe influenza diseases from Taiwan CDC and local data in a university-affiliated hospital in southern Taiwan, 2019–2020	*							
29	Young [34]	Letter to the editor	China, Italy and the USA	influenza incidence was obtained from the WHO website from 2015 to 2019 vs. 2020	*							

This study has several limitations. First, it has been more than a year since the beginning of the COVID-19 pandemic; however, many aspects are still unknown and limited studies are available regarding the effect of the COVID-19 prevention measures on the spread of other respiratory infections. Second, many studies came from specific parts of the world; for example, Southeast Asia is responsible for a significant part of the studies discussed in our review. Therefore, the knowledge of the various endemic patterns related to other regions in the world is limited and we were not able to discuss it in detail. At last, novel studies might contradict the findings of our study as the knowledge on the current pandemic and its effects are rapidly evolving worldwide. Overall, this study presented novel findings on the effect of the COVID-19 prevention measures on several infections and provided valuable information to conduct future studies.

## Conclusion

In conclusion, most of the studies suggested that the implemented preventive COVID-19 protocols have controlled and reduced the outbreaks of influenza and several other respiratory infectious diseases. The impact of the COVID-19 pandemic and the prevention measures on other respiratory infections could be attributed to an increase in positive testing, reduction in the patients with these infectious diseases, or in some cases, it might be due to the increased mortality of these diseases. However, the overall findings indicate the positive effect of COVID-19 preventive measures on controlling the seasonal endemics of other respiratory diseases; even though controversies still exist, and further studies are needed to clarify the exact impact of the COVID-19 on other respiratory infections.

## Data Availability

The authors stated that all information provided in this article could be shared. Ethics approval and consent to participate The present study was extracted from the research project with code IR.KHALUMS.REC.1399.001 entitled "Investigation of effective drugs for people affected by Coronavirus disease 2019 (COVID-19) in Imam Khomeyni hospital" conducted at Khalkhal University of Medical Sciences in 2020.
